# Association of Serum Lipids with 10-Year CVD and All-Cause Mortality in Iranian Adults: A Prospective Cohort Study

**DOI:** 10.34172/aim.34909

**Published:** 2025-12-01

**Authors:** Maryam Saberi-Karimian, Maryam Mohammadi-Bajgiran, Niloofar Shabani, Farima Farsi, Sara Saffar Soflaei, Farnaz Farrokhzadeh, Hanieh Keikhay Moghadam, Habibollah Esmaily, Mohsen Moohebati, Gordon A. Ferns, Mahmoud Ebrahimi, Majid Ghayour-Mobarhan

**Affiliations:** ^1^Metabolic Syndrome Research Center, Mashhad University of Medical Sciences, Mashhad, Iran; ^2^International UNESCO Center for Health-Related Basic Sciences and Human Nutrition, Mashhad University of Medical Sciences, Mashhad, Iran; ^3^Department of Biostatistics, School of Health, Tehran University of Medical Sciences, Tehran, Iran; ^4^Obesity and Eating Habits Research Institute, Endocrinology and Metabolism Clinical Sciences Research Institute, Tehran University of Medical Sciences, Tehran, Iran; ^5^Department of Biostatistics, School of Health, Mashhad University of Medical Sciences, Mashhad, Iran; ^6^Social Determinants of Health Research Center, Mashhad University of Medical Sciences, Mashhad, Iran; ^7^Heart and Vascular Research Center, Mashhad University of Medical Sciences, Mashhad, Iran; ^8^Department of Cardiology, Faculty of Medicine, Mashhad University of Medical Sciences, Mashhad, Iran; ^9^Brighton and Sussex Medical School, Division of Medical Education, Brighton, UK; ^10^Vascular and Endovascular Research Center, Faculty of Medicine, Mashhad University of Medical Sciences, Mashhad, Iran

**Keywords:** Cancer, Cardiovascular diseases, Cohort study, Dyslipidemia, Lipids, MASHAD, Mortality, Non-HDL Cholesterol

## Abstract

**Background:**

Individuals with abnormal serum lipid levels are at an augmented risk of atherosclerotic cardiovascular diseases (CVDs). The purpose of this study was to evaluate the significance of serum lipid concentrations as determinants for the risk of CVD and all-cause mortality (ACM).

**Methods:**

This prospective cohort study involved individuals who were part of the Mashhad stroke and heart atherosclerotic disorder (MASHAD) study initiated in 2007. A total of 9704 individuals aged 35- 65 years were involved in the current study. The participants were monitored for about a decade to track mortality and its underlying causes. Multivariable Cox proportional hazards models were applied to estimate hazard ratios (HRs) for serum levels of LDL-C, HDL-C, non-HDL-C, and triglycerides (TG), analyzed both as continuous variables and categorized into tertiles. Three models were developed: Model 1 (unadjusted), Model 2 (adjusted for age and sex), and Model 3 (further adjusted for BMI, smoking status, diabetes, hypertension, CVD, job, marital status, education level, and lipid-lowering drugs use). Kaplan–Meier survival analysis compared outcomes across lipid tertiles. Subgroup analyses were also performed to evaluate and control for confounding variables related to serum lipid levels and mortality.

**Results:**

Over a follow-up period of 10 years, there were 429 (4.4%) deaths, including 185 cases due to CVD and 124 cases due to cancer. LDL-C, HDL-C, non-HDL cholesterol, and TG were categorized into three groups based on tertiles. Based on Cox model analysis, after full adjustment, individuals in the second (37.9–45.8 mg/dL) and third (45.8–96.2 mg/dL) tertiles of HDL-C had a significantly lower risk of ACM compared with the lowest tertile (≤37.9 mg/dL) (HR=0.72, 95% CI: 0.57–0.92; and HR=0.81, 95% CI: 0.64–1.03, respectively). Similarly, the risk of cardiovascular mortality was reduced in the second tertile (HR=0.66, 95% CI: 0.46–0.94). No significant associations were found between LDL-C and mortality after adjustment. Kaplan–Meier analyses confirmed significant survival differences across HDL-C (*P* value=0.005), TG (*P* value=0.001), and non-HDL-C (*P* value<0.001) tertiles for ACM event. Significant differences were also observed in the Kaplan–Meier curves for cardiovascular death between HDL-C (*P* value=0.003) and TG groups (*P* value=0.015). The survival curves of HDL-C groups were significantly variable in terms of cancer mortality (*P* value=0.048). In exploratory subgroup analyses, the inverse correlation between elevated HDL-C levels and mortality was predominantly more pronounced in older people and those with hypertension or diabetes, whereas it was less significant in younger and healthier individuals.

**Conclusion:**

Abnormal levels of serum lipids, specifically low HDL-C concentration, are associated with an elevated risk of both non-CVD and CVD mortality. These relationships were widely seen across clinical categories, exhibiting substantially greater patterns in older participants and in persons with hypertension or diabetes. These findings indicate that HDL-C may assist in identifying individuals at increased mortality risk within this population.

## Introduction

 Non-communicable diseases (NCDs) overall, among which cardiovascular disorders (CVDs) and cancers are the major contributors, account for the large majority of deaths in Iran, with NCDs responsible for roughly three-quarters of deaths in some national summaries.^1^ Several reports project that CVDs will remain a dominant component of the disease burden through 2025 and beyond, driven by demographic aging and persistent metabolic risk factors such as high systolic blood pressure and abnormal lipid profile.^2^ Despite the global statistics, numbers may be different in smaller populations. In certain groups, like Caucasians, reduced triglycerides (TG), total cholesterol (TC), low-density lipoprotein cholesterol (LDL-C), and elevated levels of high-density lipoprotein cholesterol (HDL-C) concentrations are typically linked to decreased mortality rates.^3-6^ However, in some populations like Asian people, low cholesterol level was linked to higher mortality.^7^ ​

 ​The overall prevalence of any dyslipidemia across large recent national and cohort studies ranges from about 68% to 83% of adults, indicating that dyslipidemia is very common in Iran​.^8,9^ Cardiovascular disease (CVD) continues to be a primary factor in premature mortality in Iran. Identifying lipid patterns linked to increased mortality risk in this population may enhance the development of locally relevant screening and risk stratification thresholds. Previous studies have assumed that serum lipids including TG, TC, and LDL-C are important risk factors for CVDs.^10^ For instance, according to a recent meta-analysis, participants in the top quartile of TC variability showed an increased risk of CVDs and all-cause mortality (ACM).^11^ Furthermore, for every 1 mg/dL increase in HDL-C, there is an estimated 2% lower risk of coronary heart disease in men and 3% lower risk in women.^12^ Iranian cohorts, including the Tehran Lipid and Glucose Study (TLGS), have found different associations between HDL-C and other lipid markers with mortality and CVD events.^13-15^ According to TLGS, a U-shaped correlation was identified between non-HDL-C and the probability of ACM.^15^ Another study noted that LDL-C was not a significant risk factor for CVD or mortality events when using cut-offs of 1.84 or 2.59 mmol/L.^16^ While some studies show significant associations, others highlight that the addition of new indices to existing risk scores might not always improve predictive ability.^17^ Additionally, generalized cut-off values may not be universally applicable due to ethnic group variances in lipid profiles of Iranian populations.^18^

 Dyslipidemia has been also responsible for the development of different types of cancers, which are another common cause of death.^19^ In countries across the economic spectrum, cancer ranks among the top causes of death worldwide. Mortality rates for different cancer types are on the rise in low- and middle-income nations, primarily attributed to the growing prevalence of smoking, obesity, and sedentary lifestyles.^20^ Several studies have explored the role of dyslipidemia in different types of cancers. According to these studies, dyslipidemia was a major risk factor for breast,^21^ prostate,^22^ and colorectal cancers.^23^ Several potential mechanisms have been proposed such as intratumoral hormone production, inducing cell proliferation through inflammation, and supporting neoplastic tissue by stores of cholesterol.^24^ While there are general studies on lipid profiles and incidence of some cancers, they do not specifically address the combination of all four lipid parameters with long-term cancer mortality in an Iranian context.^25^

 In this article, we explored the connections between baseline serum lipid levels categorized into data-driven tertiles and their 10-year risks of all-cause, cardiovascular, and cancer mortality. Furthermore, these associations were examined across clinically relevant subgroups. This study’s goal is to offer population-specific epidemiologic information that may help future research on risk stratification in Iran by investigating the links between lipid parameters and long-term mortality outcomes.

## Materials and Methods

###  Study Design and Population

 The Mashhad stroke and heart atherosclerotic disorder (MASHAD) prospective cohort study, started in 2007^26^ and with follow-ups performed every three years over 10 years.

 A total of 9704 subjects aged 35 to 65 years were enlisted in the current study. The participants underwent medical history taking and laboratory testing. Serum levels of TC, HDL-C, and TG were measured using commercial kits. LDL-C was measured using the Friedewald formula.^27^ Dyslipidemia was defined as follows: serum LDL-C level ≥ 130 mg/dL, TC ≥ 200 mg/dL, TG ≥ 150 mg/dL, or HDL-C levels < 40 mg/dL in males and < 50 mg/dL in females.^28^

###  Study Endpoint and Follow-up

 The study population was followed over an average follow-up period of approximately 10 years. To minimize the possibility of losing contact, they were contacted every three years, during which they were asked to fill out follow-up surveys on how their health and lifestyle had changed. The death cause questionnaire was completed by the participant’s spouse or children. Moreover, the cause of death was extracted from the death registry of the Iranian Ministry of Health based on ICD-10 codes. We searched for death causes in the death registry using the national IDs of the participants as well as their name, father’s name and birth date. ICD codes considered as CVD death are listed in Supplementary file 1 (Table S1). In case of discrepancy in the cause of death from the two above sources, the cause was determined by the committee containing at least a cardiologist and a general practitioner. The date of last follow-up was considered the time of death for the dead participants and the date of recruiting for phase 2 of MASHAD study for alive subjects. The classification of cardiovascular deaths followed a physician review consistent with prior MASHAD cohort publications.^29-31^

###  Statistical Analysis

 Microsoft Visual Studio was used to transfer the field centers to the data bank. The NET software is only intended for Mashhad study data management. Data analysis was done using R version 4.3.2 and SPSS version 27, employing two-tailed tests for all analyses. Descriptive data such as mean, frequency, and standard deviation (SD) were used to describe the data. Depending on the distribution of quantitative data, an appropriate parametric or non-parametric test was used. The chi-square test was used to analyze qualitative variables. Kaplan-Meier curves were used to evaluate survival differences across different serum lipid level groups. We used three hierarchical Cox proportional-hazards models to assess the association of serum lipid levels with mortality. Model 1 was unadjusted. Model 2 was a minimally adjusted model including age (years, continuous) and sex (male/female). Model 3 was the fully adjusted model and included the variables in Model 2 plus the following *a priori* covariates: body mass index (BMI, kg/m^2^, continuous), smoking status (never/former/current), diabetes (yes/no), hypertension (yes/no), history of CVD (yes/no), lipid-lowering medication use at baseline (yes/no), education level (illiterate/school/college), occupation (employed/unemployed/retired/student), and marital status (single/divorced/married/widowed). In Model 3, lipid-lowering medication use was included as a covariate to adjust for its potential influence on mortality outcomes. Information on dosage, duration, or specific timing of treatment was not available.

 Covariates were chosen *a priori* based on the literature^32^ showing them as potential confounders of the association between lipid levels and mortality (they are associated with both exposures (lipids) and outcomes (all-cause and cardiovascular mortality)) and on causal reasoning. We did not use automated (stepwise or data-driven) variable selection; instead, model covariates were prespecified. Models were built sequentially (Model 1 → Model 2 → Model 3) to show the influence of incremental adjustment on the exposure–outcome associations. All covariates were included in Model 3 simultaneously. The proportional hazards assumption was tested for all Cox models using Schoenfeld residuals (no major violations were observed).

 Missing data were handled using a complete-case approach (subjects with missing values for covariates in a given model were excluded from that model). A significance level of *P* value < 0.05 was applied to all comparisons.

 No formal *a priori* sample-size or power calculation for specific hazard ratios (HRs) was performed because this was an observational analysis of the existing MASHAD cohort. To assist in interpretation of HRs, we performed a *post-hoc* detectable-effect calculation using the Schoenfeld approximation for Cox models (two-sided α = 0.05, power = 80%).

 This analysis was exploratory and was conducted without formal adjustment for multiple comparisons. Although performing multiple hypothesis tests increases the risk of type I error, we selected not to apply corrections such as Bonferroni or false discovery rate (FDR) adjustments in order to preserve sensitivity for detecting potential trends. For context, a Bonferroni correction for 36 tests (2 outcomes × 4 exposures × 3 models = 24) would yield a significance threshold of approximately *P* < 0.002. However, this adjustment was not applied in the current exploratory analyses. Accordingly, these findings should be considered hypothesis-generating and require confirmation in future studies.

 Time-dependent receiver operating characteristic (ROC) analyses were performed using predicted survival probabilities from the fully adjusted Cox proportional-hazards model (Model 3). The time-dependent AUC was estimated at the median follow-up time (121.2 months) to assess model discrimination at that time point. The optimal HDL-C threshold was defined as the value maximizing the Youden index (J = sensitivity + specificity – 1). Sex-stratified ROC analyses were also performed to obtain optimal cut-offs for men and women separately. Time-dependent ROC and AUC calculations were conducted using the ‘timeROC’ package in R (version 4.3.2).

## Results

###  Comparison of Participants’ Characteristics and Serum Lipid Levels by Cardiovascular Death

 Among 9704 individuals included in the study, 3885 were male and 5819 were female. Over the 10-year follow-up period, 185 (1.91%) deaths due to CVD were recorded. Participants who experienced cardiovascular deaths were older than those who did not. Additionally, individuals with cardiovascular death had a higher prevalence of hypertension, diabetes, and CVD compared to those without cardiovascular death. The baseline characteristics and serum lipid levels of the participants are detailed in Table 1.

**Table 1:** Baseline characteristics of the population with 1–10-year survivors and CVD mortality

		**1–10-year survivors (n=9519)**	**CVD mortality (n=185)**	***P*** ** value**
Age (y)		47.95 (8.22)	54.79 (7.58)	< 0.001*
Age group (y), n (%)	< 48	9284 (97.6)	173 (94)	0.002*
	≥ 48	230 (2.4)	11 (6)	
Gender, n (%)	Male	3769 (39.6)	116 (62.7)	< 0.001*
	Female	5750 (60.4)	69 (37.3)	
Marriage, n (%)	Single or divorced	190 (2)	3 (1.6)	0.879
	Married	8864 (93.1)	174 (94.1)	
	Widowed	464 (4.9)	8 (4.3)	
Job, n (%)	Student or employed	3558 (37.4)	67 (36.2)	< 0.001*
	Unemployed	5041 (53)	84 (45.4)	
	Retired	916 (9.6)	34 (18.4)	
Education, n (%)	Illiterate	1058 (11.1)	39 (21.1)	< 0.001*
	School education	7004 (73.8)	124 (67)	
	University education	1431 (15.1)	22 (11.9)	
Smoking status, n (%)	Non-smoker	6556 (68.9)	98 (53)	< 0.001*
	Ex-smoker	926 (9.7)	32 (17.3)	
	Current smoker	2037 (21.4)	55 (29.7)	
Anti lipid agents		135 (1.4)	6 (3.2)	0.040*
Insulin		19 (0.2)	5 (2.7)	< 0.001*
Anti HTN agents		342 (3.6)	12 (6.5)	0.038*
Hypertension, n (%)		2201 (23.2)	93 (50.3)	< 0.001*
Diabetes, n (%)		1298 (13.8)	71 (39.2)	< 0.001*
Dyslipidemia, n (%)		8096 (85.6)	156 (85.7)	0.965
CVD, n (%)		192 (2)	17 (9.2)	< 0.001*
TC (mg/dL)		191.17 (39.03)	199.55 (44.56)	0.004*
TG (mg/dL)		120 (87)	136.5 (130)	0.007*
HDL-C (mg/dL)		42.88 (9.95)	40.92 (9.75)	0.009*
Non-HDL-C (mg/dL)		148.29 (37.16)	158.62 (41.95)	< 0.001*
LDL-C (mg/dL)		116.49 (35.19)	121.17 (37.51)	0.077

###  Correlation between Serum Lipid Levels and CVD, Cancer, and All-cause Mortality

 Total follow-up throughout the study was 93,465.7 person-years (PY) (mean follow-up ≈9.85 years). Person-years by LDL-C tertile were 29,547.5 (N = 3,165), 31,746.1 (N = 3,193), and 32,172.1 (N = 3,282). Overall mortality corresponded to 4.59 deaths per 1,000 person-years (429 deaths/93,465.7 PY), and cardiovascular mortality to 1.98 deaths per 1,000 person-years (185 deaths/93,465.7 PY). Cause-specific event counts and person-years by tertile are shown in Table 2.

**Table 2:** Univariable and Multivariable Cox Regression Analyses of LDL-C, HDL-C, non-HDL-C, and TG Association with Mortality

		**Range of tertiles & N-at-risk**	**Person-years**	**All-cause mortality (ACM) (n=429)**			**CVD mortality (n=185)**		
				**Number of events**	**HR (95%CI)** ***P *** **value**	***P *** **value -Trend**	**Number of events**	**HR (95%CI)** ***P *** **value**	***P*** ** value -Trend**
Model 1	LDL-C group (mg/dL)	[12.2,100.8) N = 3165	29547.49	128	Ref	0.041*	51	Ref	0.031*
		[100.8,128.7) N = 3193	31746.08	132	1.06 (0.83,1.35) 0.658		55	1.10 (0.75,1.61) 0.628	
		[128.7,303.4] N = 3282	32172.13	162	1.27 (1.01,1.60) 0.043*		75	1.47 (1.03,2.09) 0.035*	
	HDL-C group (mg/dL)	[12.8,37.9) N = 3146	30907.88	173	Ref	< 0.001**	80	Ref	0.002*
		[37.9,45.8) N = 3195	31440.40	122	0.70 (0.55,0.88) 0.002 *		51	0.63 (0.44,0.89) 0.009*	
		[45.8,96.2] N = 3298	32684.83	128	0.68 (0.54,0.86) 0.001**		51	0.59 (0.41,0.84) 0.003*	
	Non-HDL-C group (mg/dL)	[23.4,130) N = 3130	30963.52	118	Ref	0.002*	48	Ref	0.017*
		[130,160.4) N = 3226	31898.04	132	1.09 (0.85.1.39) 0.517		58	1.17 (0.8,1.72) 0.414	
		[160.4,392.20] N = 3282	32163.93	173	1.43 (1.13,1.80) 0.003*		76	1.54 (1.07,2.21) 0.019*	
	TG group (mg/dL)	[21,95) N = 3132	31030.92	117	Ref	< 0.001**	52	Ref	0.017*
		[95,149) N = 3186	31475.77	128	1.08 (0.84,1.39) 0.538		50	0.95 (0.64,1.40) 0.447	
		[149,1225] N = 3322	32535.33	178	1.48 (1.17,1.87) < 0.001**		80	1.50 (1.05,2.12) 0.024*	
Model 2	LDL-C group (mg/dL)	[12.2,100.8) N = 3165	29547.49	128	Ref	0.277	51	Ref	0.078
		[100.8,128.7) N = 3193	31746.08	132	1.01 (0.79,1.29) 0.924		55	1.06 (0.72,1.55) 0.777	
		[128.7,303.4] N = 3282	32172.13	162	1.13 (0.90,1.43) 0.288		75	1.37 (0.96,1.96) 0.087	
	HDL-C group (mg/dL)	[12.8,37.9) N = 3146	30907.88	173	Ref	0.025*	80	Ref	0.056
		[37.9,45.8) N = 3195	31440.40	122	0.75 (0.59,0.95) 0.016*		51	0.71 (0.50,1.01) 0.059	
		[45.8,96.2] N = 3298	32684.83	128	0.77 (0.61,0.98) 0.031*		51	0.71 (0.49,1.03) 0.070	
	Non-HDL-C group (mg/dL)	[23.4,130) N = 3130	30963.52	118	Ref	0.216	48	Ref	0.174
		[130,160.4) N = 3226	31898.04	132	0.96 (0.74,1.23) 0.722		58	1.04 (0.71,1.53) 0.828	
		[160.4,392.20] N = 3282	32163.93	173	1.15 (0.90,1.45) 0.255		76	1.27 (0.88,1.84) 0.193	
	TG group (mg/dL)	[21,95) N = 3132	31030.92	117	Ref	0.106	52	Ref	0.227
		[95,149) N = 3186	31475.77	128	0.97 (0.75,1.24) 0.779		50	0.86 (0.58,1.27) 0.447	
		[149,1225] N = 3322	32535.33	178	1.20 (0.95,1.51) 0.134		80	1.21 (0.85,1.72) 0.288	
Model 3	LDL-C group (mg/dL)	[12.2,100.8) N = 3165	29547.49	128	Ref	0.741	51	Ref	0.279
		[100.8,128.7) N = 3193	31746.08	132	0.95 (0.74,1.21) 0.667		55	0.99 (0.67,1.46) 0.970	
		[128.7,303.4] N = 3282	32172.13	162	1.04 (0.82,1.31) 0.767		75	1.21 (0.84,1.74) 0.299	
	HDL-C group (mg/dL)	[12.8,37.9) N = 3146	30907.88	173	Ref	0.069	80	Ref	0.082
		[37.9,45.8) N = 3195	31440.40	122	0.72 (0.57,0.92) 0.007*		51	0.66 (0.46,0.94) 0.023*	
		[45.8,96.2] N = 3298	32684.83	128	0.81 (0.64,1.03) 0.092		51	0.74 (0.51,1.08) 0.119	
	Non-HDL-C group (mg/dL)	[23.4,130) N = 3130	30963.52	118	Ref	0.892	48	Ref	0.811
		[130,160.4) N = 3226	31898.04	132	0.94 (0.73,1.21) 0.620		58	1.02 (0.69,1.51) 0.912	
		[160.4,392.20] N = 3282	32163.93	173	1.01 (0.79,1.28) 0.943		76	1.05 (0.72,1.52) 0.812	
	TG group (mg/dL)	[21,95) N = 3132	31030.92	117	Ref	0.875	52	Ref	0.682
		[95,149) N = 3186	31475.77	128	0.91 (0.71,1.18) 0.502		50	0.75 (0.50,1.12) 0.161	
		[149,1225] N = 3322	32535.33	178	1.01 (0.78,1.29) 0.961		80	0.89 (0.61,1.30) 0.546	

Model 1: Unadjusted. Model 2: Adjusted for age group and sex. Model 3: Adjusted for age group, sex, BMI, smoking status, diabetes, hypertension, CVD, job, marriage status, education level, and lipid-lowering drugs. Note: Given that 2 outcomes, 4 exposures, and 3 models were tested (24 total comparisons), the Bonferroni-corrected significance threshold would be *P* < 0.002. *P *values above this threshold should be interpreted with caution, as these analyses are exploratory and were not adjusted for multiple comparisons. Results significant at *P* < 0.05 are marked with *, while results that remain significant after Bonferroni correction are marked with **. Note: Analyses are based on complete cases.

 The relationship between serum lipid levels and cause-specific mortality as well as ACM is presented in Table 2. Initially, in the unadjusted Model 1, LDL-C, HDL-C, non-HDL-C, and TG were considered as quantitative variables. A significant correlation was found between HDL-C, non-HDL-C, and TG with ACM and CVD mortality. Subsequently, LDL-C, HDL-C, non-HDL-C, and TG were categorized into tertiles and included in the unadjusted Cox model. It was observed that the risk of ACM and CVD mortality in the third tertile of LDL-C, non-HDL-C, and TG was significantly higher than the first tertile. Moreover, the risk of ACM and CVD mortality in the second and third tertiles of HDL-C was significantly lower than in the first tertile of HDL-C (Table 2).

 In Model 2, after adjusting for age and sex, individuals in the second tertile of HDL-C had a significantly lower risk of ACM compared with those in the first tertile (HR = 0.75, 95% CI: 0.59–0.95; *P* = 0.016), and those in the third tertile also showed a reduced risk (HR = 0.77, 95% CI: 0.61–0.98; *P* = 0.031) (Table 2). In Model 3, which further adjusted for BMI, smoking status, diabetes, hypertension, CVD, occupation, marital status, education level, and use of lipid-lowering medications, the protective association remained consistent. Specifically, in Model 3, individuals in the second tertile of HDL-C had a lower risk of ACM (HR = 0.72, 95% CI: 0.57–0.92; P = 0.007) and CVD mortality (HR = 0.66, 95% CI: 0.46–0.94; P = 0.023), although the latter did not reach statistical significance.

**Figure 1:**
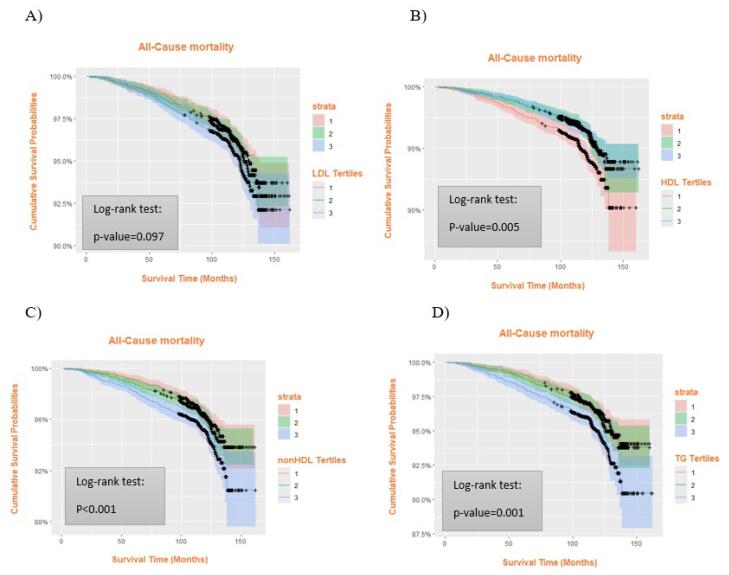
Comparison of All-cause Mortality between Serum Lipid Tertiles in Kaplan-Meier Curves. A) LDL, B) HDL-C, C) non-HDL-C, and D) TG. High serum lipid levels except for LDL-C, increased the all-cause mortality risk. Tertiles represent low (T1), moderate (T2), and high (T3) levels of each lipid profile. Colored shaded areas indicate 95% confidence intervals (CIs): pink for T1, green for T2, blue for T3, and purple for overlapping CIs. + shows censored cases. LDL: low-density lipoprotein; HDL: high-density lipoprotein; TG: triglycerides

**Figure 2:**
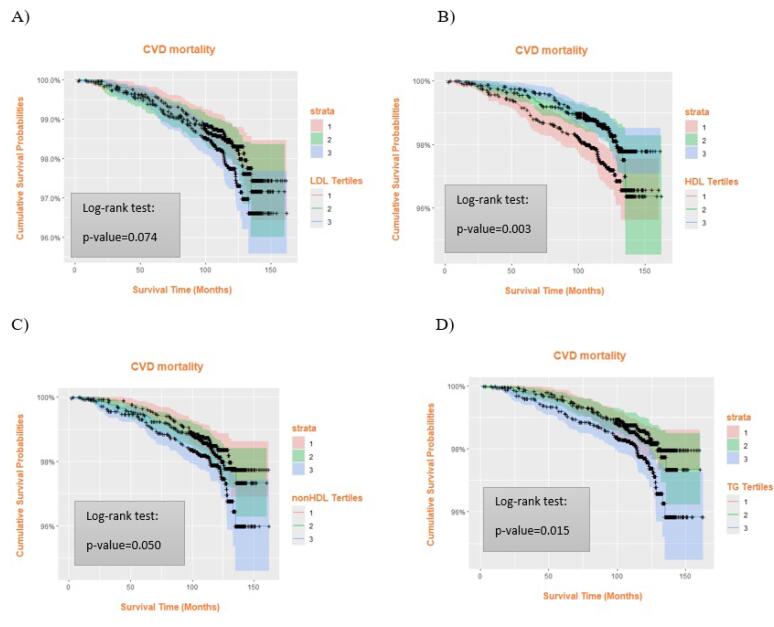
Comparison of CVD Mortality between Different Serum Lipid Tertiles. A) LDL, B) HDL-C, C) non-HDL-C, and D) TG. High TG and low HDL-C levels were associated with an increased risk of CVD mortality. Tertiles represent low (T1), moderate (T2), and high (T3) levels of each lipid profile. Colored shaded areas indicate 95% confidence intervals (CIs): pink for T1, green for T2, blue for T3, and purple for overlapping CIs. + shows censored cases. CVD: cardiovascular disorders; LDL: low-density lipoprotein; HDL: high-density lipoprotein; TG: triglycerides

**Figure 3:**
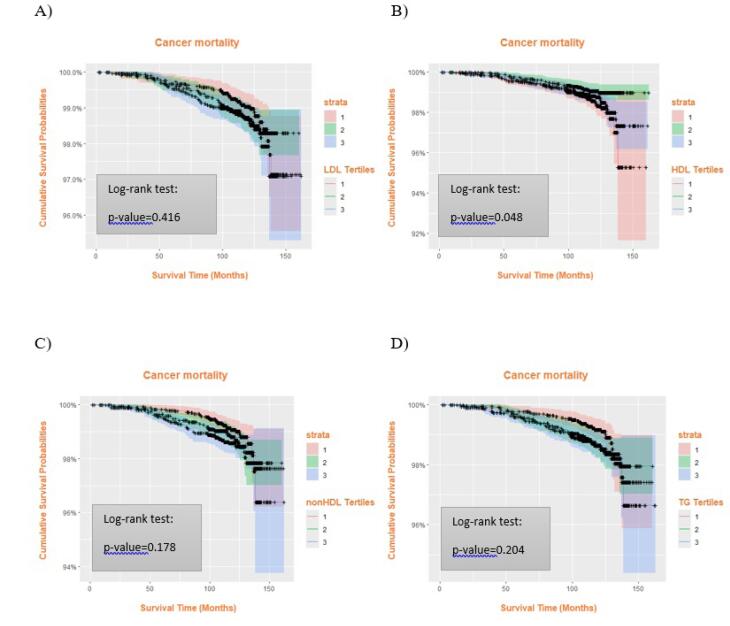
Comparison of Cancer Mortality between Serum Lipid Tertiles. Subjects with low HDL-C levels were more susceptible to dying because of cancer. A) LDL, B) HDL-C, C) non-HDL-C, and D) TG. Tertiles represent low (T1), moderate (T2), and high (T3) levels of each lipid profile. Colored shaded areas indicate 95% confidence intervals (CIs): pink for T1, green for T2, blue for T3, and purple for overlapping CIs. + shows censored cases. LDL: low-density lipoprotein; HDL: high-density lipoprotein; TG: triglycerides

 As illustrated in Figure 1, the association between different tertiles of HDL-C, non-HDL-C, and TG with ACM was significant based on the log-rank test, whereas LDL-C tertiles showed no notable difference in ACM (Figure 1). In Figure 2, Kaplan–Meier curves demonstrated significant differences in cardiovascular mortality across HDL-C tertiles (*P* = 0.003) and TG tertiles (*P* = 0.015). Additionally, Figure 3 shows that HDL-C tertiles were significantly associated with cancer mortality (*P* = 0.048).

 Subgroup analyses, summarized from Supplementary file 1 (Figure S1), indicated that the inverse association between HDL-C levels and ACM or CVD mortality was generally consistent across most demographic and clinical categories, although the strength of the association varied. In participants aged ≥ 48 years, the risk of ACM was significantly decreased in the second tertile of HDL-C category (HR = 0.73, 95% CI: 0.56–0.95) and CVD mortality (HR = 0.58, 95% CI: 0.38–0.89), while among those < 48 years, the association was weaker and not statistically significant. Both men and women showed a similar protective pattern of higher HDL-C, with no significant HDL-C × sex interaction.

 Participants with pre-existing diabetes or hypertension exhibited a stronger inverse association between HDL-C and mortality risk compared to those without these comorbidities. For example, in participants with diabetes, the HR for ACM in the lowest HDL-C tertile versus the highest was 0.53 (95% CI: 0.34–0.84), while among participants without diabetes, the association was attenuated (HR = 0.98, 95% CI: 0.73–1.31). Several subgroup analyses, particularly those for CVD outcomes, were based on a small number of events. This resulted in wide confidence intervals and unstable HR estimates. Consequently, some findings (such as seeming associations in CVD subgroups) may be imprecise. We therefore emphasize that these subgroup results should be interpreted with caution.

 Overall, higher HDL-C levels were associated with a lower risk of mortality, particularly among individuals aged > 48 years and those with diabetes or hypertension. However, some subgroup effects were borderline and should be interpreted cautiously.

 Because multiple comparisons were performed, statistically significant findings should be interpreted cautiously. We focus on overall patterns of association and consistency across models rather than individual *P*-values alone.

 Furthermore, time-dependent ROC curve analyses (Figure S2) were conducted based on the fully adjusted Model 3 to evaluate the prognostic performance of HDL-C for mortality outcomes. The time-dependent AUC, estimated at the median follow-up time of 121.2 months, was 0.509 in men and 0.534 in women for ACM, and 0.512 in men and 0.578 in women for cardiovascular mortality, indicating modest discrimination. The optimal HDL-C cut-off points, determined using the maximum Youden index, were 37.6 mg/dL for men and 40.0 mg/dL for women for ACM, and 34.1 mg/dL for men and 40.0 mg/dL for women for cardiovascular mortality (Figure S2). The number of subjects at risk and the estimated 5-year and 10-year survival probabilities across lipid profile tertiles are presented in Supplementary file 1 (Table S2).

## Discussion

 Dyslipidemia refers to elevated concentration of TG, TC, LDL-C, and decreased concentration of HDL-C. Among the world’s leading causes of long-term disability and death, coronary artery disease (CAD) and stroke as well as other vascular disorders associated with atherosclerosis, dyslipidemia plays a major role in their development.^24,33^

 In this study, higher HDL-C concentrations with a significant reflection point of 37.9 mg/dL were associated with a lower probability of all-cause, CVD, and cancer mortality. Moreover, high serum levels of TG ( > 149 mg/dL) were associated with higher ACM and CVD mortality risk; however, this increase was not statistically significant. Additionally, people with elevated non-HDL cholesterol levels ( > 160.4 mg/dl) were more likely to develop ACM. Although LDL-C had no significant relationship with all-cause, CVD, and cancer mortality, when estimated with other underlying parameters such as age, gender, hypertension, and dyslipidemia, it could affect the mortality rate. These results indicate that reduced HDL-C and elevated triglyceride and non-HDL-C levels correlated with adverse mortality outcomes, with these trends potentially being more pronounced in older individuals and those with comorbidities like hypertension. But these subgroup links were based on a small number of cardiovascular deaths and had wide confidence intervals. Consequently, these subgroup results should be regarded as hypothesis-generating rather than conclusive, necessitating validation in larger cohorts with enhanced statistical power.

 This is the first study to investigate the relationship between different serum levels of all lipoproteins in a large cohort over an extended period of follow-up with comprehensive phenotype data including conditions linked to biological aging and morbidity, as well as cardiovascular and non-cardiovascular outcomes. Although the cohort size was substantial, some participants were excluded due to missing baseline laboratory or covariate data, and medication use was recorded only as a binary baseline variable, which may introduce selection bias and limit treatment adjustments.

 Epidemiological studies have identified a lipid paradox present in diverse populations. While the link between LDL-C levels and mortality risk in various countries remained a topic of debate, numerous studies have established LDL-C as a primary factor in the development of atherosclerotic disease, and current prevention guidelines continue to target LDL-C aggressively for atherosclerotic cardiovascular disease (ASCVD) risk reduction, with recommended LDL-C goals as low as < 55 mg/dL in very high-risk individuals.^34,35^ It is widely acknowledged that lipid-lowering treatment is crucial to prevent ASCVD events, although recent analyses suggest that the survival benefit of statin therapy may not be explained solely by the degree of LDL-C reduction, implying possible pleiotropic effects beyond cholesterol lowering.^36,37^ In this investigation, we observed a significant association between LDL-C concentration and ACM and CVD mortality with an inflection point of 128.7 mg/dL. However, after adjusting for baseline characteristics, no association between LDL-C and mortality rate was observed, while HDL-C was found to be protective. This pattern is consistent with reports that LDL-C is not always linearly associated with ACM in general or treated populations, and that the so-called “cholesterol paradox”, in which lower LDL-C appears linked to higher mortality, may partly reflect reverse causation and population differences.^38,39^ Even though LDL-C did not have a clear link to death rates, the difference in CVD death rates between LDL-C tertiles in our study was small (about 0.5 deaths per 1,000 person-years) so it probably does not have much of an effect on the population as a whole. This correlation was not with non-CVD mortality, but with CVD death. A similar relationship was reported by the MONDO study, where higher LDL-C concentration was linked to lower risk of non-cardiovascular death in patients with end-stage renal disease but not with CVD mortality.^40^ Low LDL-C was also strongly associated with increased mortality from all causes except CVD problems, as shown in a cohort study among a Danish population.^41^ Another investigation demonstrated that low LDL-C had a synergistic effect with HDL-C on cardiovascular mortality in critically ill patients.^42^ In contrast, according to a study by Peng et al, which was a meta-analysis of 20 cohort studies, LDL-C ≥ 130 mg/dL was associated with augmented risk of ACM and CVD risk.^43^ Similarly, according to a study by Nguyen et al, reverse causality was evident in the cholesterol paradox, which refers to the higher risk of coronary heart disease death in individuals with low cholesterol concentrations, particularly in older subjects. Reduced CVD death was therefore linked to lower blood cholesterol.^44^ In a prospective cohort study on the USA general population, it was determined that increased risks of CVD mortality were linked to both extremely high (LDL-C ≥ 190 mg/dL) and low (LDL-C < 70 mg/dL) LDL-C levels. Moreover, very low serum LDL-C concentration was also related to high risks of stroke and ACM.^45^

 The detrimental consequences of both low and high LDL-C levels could be explained by several factors. LDL-C contributes to forming plaques and therefore reducing blood flow through coronary vessels to myocardial cells, which is known as ischemia. Ischemia rapidly lowers systolic function and causes significant metabolic and ionic disruptions in the affected myocardium, making it susceptible to future adverse cardiac events.^46^ On the other hand, transduction of intracellular signal pathways depends critically on cholesterol, an essential component of cell membranes. It is also a substrate for the synthesis of steroid hormones, which helps the body withstand lethal stress. Moreover, LDL-C plays a protective role in the body’s immune response to various pathogens. Consequently, very low concentrations of LDL-C may heighten the risk of severe illnesses.^47^

 Our research indicated that maintaining HDL-C levels above 37.9 mg/dL correlated with lower risk of mortality from various causes, including ACM, cancer, and cardiovascular death. Likewise, Mørland et al found a notable relationship between lower HDL-C levels and higher mortality rates related to CVDs, gastric cancer, and diabetes.^48^ However, recent large cohort and meta-analysis data suggest that this association follows a J-shaped or U-shaped pattern, where both very low and very high HDL-C concentrations are linked to elevated mortality risks.^49-51^ For instance, Mørland et al discovered that mortality rates for conditions such as alcoholic liver disease, chronic liver diseases, cancers of the mouth, esophagus, and liver, accidents, diabetes, and chronic obstructive pulmonary disease rose individually when HDL-C levels exceeded 50-59 mg/dL. According to studies from CANHEART and pooled analyses, the lowest ACM risk was observed at intermediate HDL-C levels (approximately 54–70 mg/dL), while extremely high levels were associated with increased non-cardiovascular mortality, particularly among men.^50,52,53^ In contrast, we did not find such a relationship between HDL-C concentrations and either ACM and CVD mortality. In our study, the second tertile of the HDL-C levels (37.9-45.8 mg/dL) compared to the reference value (12.8-37.9 mg/dL) had a significantly lower mortality rate for all reasons even after adjustment for baseline characteristics and lipid-lowering drugs. However, as illustrated in Figure 3, the curves for the lowest (12.8-37.9 mg/dL) and highest tertiles (45.8-96.2 mg/dL) of HDL-C dropped more steeply compared to the middle tertile. In other words, there was a U-shaped association between HDL-C levels and cancer mortality. Similarly, according to Halsey et al, the ratio of total/HDL-C was the strongest predictor of IHD mortality and there was an inverse association between HDL-C levels up to 70 mg/dL and IHD mortality, but not for stroke death.^54^ Other studies have also confirmed that low HDL-C concentrations cause an increased risk of CVD mortality more pronounced for IHD and other CVDs rather than stroke.^50,53^ Our findings were not in concert with a study on the Danish population reporting increased CVD mortality for subjects with HDL-C concentrations above 90–100 mg/dL.^55^ Overall, evidence from multiple large cohorts supports a J-shaped association between HDL-C and mortality from various causes, suggesting that both excessively high and low HDL-C concentrations may confer increased risk.^50^ Recent reviews also emphasize that HDL functionality rather than HDL-C concentration alone may better capture cardioprotective effects.^56,57^

 Based on our study results, a significant relationship between high non-HDL cholesterol concentrations above 160.4 mg/dL and TG concentrations of more than 149 mg/dL and both ACM and CVD mortality was observed. However, after adjustment for confounding variables including age, hypertension, and dyslipidemia, the relationship did not persist. High TG levels are associated with atherosclerosis, which may contribute to increased risk of stroke and adverse cardiac events. Moreover, extremely high TG can cause acute pancreatitis.^58^ Likewise, a cohort study by Klempfner et al found that for individuals with CAD, every one unit increase in natural logarithm (Ln) TGs was linked to a 6% rise in the risk of ACM over a 22-year period, even when TG serum levels ranged from 100-149 mg/dL.^59^ Our findings differ from a prospective cohort study on the Chinese population, which demonstrated that people with higher amounts of TG levels ( ≥ 193.9 mg/dL) had lower risk of ACM and CVD mortality.^60^ Several constituents of non-HDL cholesterol have been reported to be atherogenic, aiding the pathophysiology of atherosclerosis. Non-HDL cholesterol represents the cholesterol content present in all the atherogenic lipoproteins (non-HDL-C = TC -HDL−C). Huang et al concluded that both very high and low non-HDL cholesterol levels were associated with higher mortality rates.^61^ Cheang et al also found a U-shaped association between non-HDL cholesterol levels and ACM; however, non-HDL cholesterol did not affect CVD mortality.^62^ Several studies have confirmed that high non-HDL cholesterol concentrations are related to increased risk of ACM and CVD mortality, which is consistent with our results. Furthermore, non-HDL cholesterol was a better predictor of future adverse cardiac events, including myocardial infarction.^63-65^ A similar result was found among patients with HTN.^66^

 Early deaths were rare in our cohort, with only 31 of 429 total deaths occurring within the first 2 years of follow-up. Given the small number of early deaths, sensitivity analyses excluding these participants were not performed, although we acknowledge that reverse causation cannot be entirely excluded.

 Furthermore, our study included multiple exposures and outcomes, which raises the possibility of false-positive findings. Consistent associations across models and with prior literature increase confidence in the robustness of our key findings; however, isolated significant results should be interpreted with caution. Even notable statistical correlations reveal minimal absolute disparities in mortality rates across lipid tertiles, indicating that substantial HRs reflect slight absolute risk variations over a decade. This underscores the necessity for population-level interventions and holistic management of risk factors, rather than concentrating exclusively on lipid levels. It should be noted that multiple comparisons increase the likelihood of false-positive findings. Because our subgroup analyses were exploratory, we did not apply corrections such as Bonferroni or FDR adjustments. Future confirmatory studies are warranted to evaluate these subgroup effects using appropriate statistical corrections.

 The current study examined a large number of participants with a relatively extended median follow-up period. Nevertheless, several limitations should be considered. Despite thorough adjustments for numerous covariates, residual confounding may persist due to unmeasured variables. We were unable to fully account for frailty, subclinical illness, or the intensity and duration of smoking exposure, and socioeconomic factors were only indirectly measured, all of which may affect lipid levels and mortality risk. In addition, the evaluation of blood lipids was limited to baseline measurements, which could have been influenced by exposures or changes occurring after study initiation, introducing possible regression dilution bias. Although longitudinal modeling could address such variability, this approach was beyond the scope of the present investigation. Since this was an observational cohort study with lipid levels measured solely at baseline, our findings indicate associations rather than causal relationships, and residual confounding or reverse causation cannot be discounted. Additional analyses are also warranted to compare the predictive value of HDL-C with that of LDL-C as a clinical indicator.

 Despite statistically significant relationships with mortality, HDL-C and other lipid markers had inadequate predictive value. HDL-C alone cannot consistently predict all-cause and cardiovascular death in time-dependent ROC studies (AUC values ~0.51–0.58 at 10-year follow-up). These biomarkers’ incremental predictive value is unknown and should be investigated in future research. We did not test calibration or if adding lipid measurements enhances prediction beyond multivariable risk scores. Moreover, while we adjusted for multiple potential confounders in the fully adjusted model to minimize residual bias, the inclusion of numerous covariates relative to the limited number of cardiovascular deaths may have reduced statistical power and widened confidence intervals. This may partly explain the attenuation of several associations that were significant in simpler models. Although multicollinearity diagnostics (variance inflation factors < 2 for all covariates) indicated no severe collinearity, some degree of overadjustment cannot be ruled out. Future studies with larger numbers of outcome events or the use of penalized regression techniques could provide more stable estimates. Additionally, these results are derived from a single regional adult cohort and may not be applicable to younger demographics, diverse ethnic groups, or environments characterized by distinct cardiovascular risk profiles or treatment modalities. Before using these findings in clinical practice, they need to be tested on other groups. In addition, cause-specific Cox models for CVD and cancer mortality may be influenced by competing risks from non-CVD or non-cancer deaths, which could bias the estimated HRs. Unfortunately, detailed data required for competing risk analyses, such as Fine–Gray models, were not available in this study, and this should be considered a limitation.

 Finally, information on several lifestyle and socioeconomic factors (such as dietary habits, physical activity, alcohol consumption, and economic status) was not available in our dataset. Additionally, data on lipid-lowering therapy were collected only as a binary variable (yes/no) at baseline, without information on dosage, treatment duration, or changes during follow-up. These limitations may have reduced the precision of our adjustments. External validation of the studied correlations in additional cohorts, mechanistic studies to determine whether HDL-C is a marker or mediator of risk, and an assessment of whether HDL-C, non-HDL-C, or TG significantly increase multivariable risk scores should be conducted in future research. Finally, intervention studies are needed to see if changing lipid patterns in certain subgroups reduces mortality.

 On the other hand, with the observed event counts (185 cardiovascular deaths; 429 all-cause deaths), the minimal detectable HR for a comparison with exposure prevalence ≈33% (tertile comparison) was approximately 1.55 for cardiovascular death and 1.33 for ACM. These calculations indicate that the study was well-suited to detect moderate-to-large associations for ACM but had limited power to detect smaller HRs for cardiovascular death. This limited power, together with adjustment for multiple covariates, may have contributed to attenuation of effects and loss of statistical significance in fully adjusted models; thus, caution is warranted when interpreting null findings for smaller effect sizes.

## Conclusion

 Our results imply that abnormal serum lipid levels, particularly low HDL-C, were associated with higher long-term risks of all-cause, cardiovascular, and cancer mortality in this adult Iranian cohort. These associations were generally modest in absolute terms and exhibited variability across clinical subgroups. Due to the observational nature of this study, the findings should be considered preliminary, necessitating external validation and interventional evidence prior to informing targeted clinical strategies.

## Supplementary Files

Supplementary file 1. Mortality Analysis
