# Supplementary material for: Association of Serum Lipids with 10-Year CVD and All-Cause Mortality in Iranian Adults: A Prospective Cohort Study
Source: Arch Iran Med. 2025 Dec 1;28(12):723–36. doi: 10.34172/aim.34909 (PMC13000341; doi:10.34172/aim.34909)
Supplement: Supplementary file 1 — Mortality Analysis [file aim-28-723-s001.pdf]

Arch Iran Med. December 2025;28(12):723-S1

10.34172/aim.34909

http://journalaim.com

Supplementary Mortality Analysis

Table S1. Recorded ICD-10 codes in the death registry, which considered a death due to cardiovascular disease.

**I20**	Angina pectoris
**I21**	Acute myocardial infarction
**I21.9**	Acute myocardial infarction, unspecified
**I25**	Chronic ischemic heart disease
**I25.1**	Atherosclerotic heart disease
**I25.9**	Chronic ischemic heart disease, unspecified
**I26.9**	Pulmonary embolism, unspecified
**I27.9**	Other pulmonary heart diseases, unspecified
**I45.1**	Atrioventricular block, complete
**I46**	Cardiac arrest
**I46.1**	Cardiac arrest due to coronary heart disease
**I49.9**	Cardiac arrhythmia, unspecified
**I50**	Heart failure
**I50.9**	Heart failure, unspecified
**I51.6**	Other specified heart disorders
**I51.9**	Heart disorder, unspecified
**I60**	Subarachnoid hemorrhage
**I60.9**	Subarachnoid hemorrhage, unspecified
**I61.5**	Hemorrhage of unspecified type, intracerebral
**I61.9**	Intracerebral hemorrhage, unspecified
**I63**	Cerebral infarction
**I63.9**	Cerebral infarction, unspecified
**I64**	Stroke, not specified as hemorrhage or infarction
**I67.8**	Other specified cerebrovascular diseases
**I67.9**	Cerebrovascular disease, unspecified
**I68.8**	Other specified disorders of cerebral vessels
**I69.4**	Sequelae of cerebral infarction
**I70.9**	Atherosclerosis, unspecified
**I80.2**	Phlebitis and thrombophlebitis of superficial veins of lower extremities

**Table S2.** Number of subjects at risk and 5-year and 10-year survival probabilities across lipid profile tertiles.				
		All cause mortality		
Index	Tertile	Number of at risk	5-year survival (95%CI)	10-year survival (95%CI)
LDL-C	T1	3113	0.990 (0.986,0.993)	0.963 (0.956,0.970)
	T2	3152	0.987 (0.983,0.991)	0.962 (0.956,0.969)
	T3	3241	0.985 (0.981,0.989)	0.954 (0.946,0.962)
HDL-C	T1	3088	0.983 (0.978,0.987)	0.948 (0.939,0.956)
	T2	3147	0.989 (0.986,0.993)	0.965 (0.959,0.972)
	T3	3270	0.990 (0.986,0.993)	0.965 (0.959,0.972)
Non HDL-C	T1	3092	0.991 (0.988,0.995)	0.964 (0.958,0.971)
	T2	3181	0.982 (0.985,0.992)	0.962 (0.956,0.969)
	T3	3231	0.982 (0.977,0.987)	0.952 (0.944,0.960)
TG	T1	3103	0.991 (0.988,0.994)	0.965 (0.958,0.972)
	T2	3147	0.989 (0.985,0.993)	0.964 (0.957,0.971)
	T3	3256	0.982 (0.978,0.987)	0.950 (0.942,0.958)
		CVD mortality		
Index	Tertile	Number of at risk	5-year survival (95%CI)	10-year survival (95%CI)
LDL-C	T1	3113	0.996 (0.994,0.998)	0.984 (0.980,0.989)
	T2	3152	0.994 (0.991,0.997)	0.984 (0.980,0.989)
	T3	3241	0.995 (0.992,0.997)	0.978 (0.972,0.983)
HDL-C	T1	3088	0.992 (0.988,0.995)	0.974 (0.968,0.980)
	T2	3147	0.996 (0.993,0.998)	0.986 (0.982,0.991)
	T3	3270	0.997 (0.995,0.999)	0.985 (0.981,0.990)
Non HDL-C	T1	3092	0.996 (0.994,0.999)	0.985 (0.981,0.990)
	T2	3181	0.995 (0.992,0.997)	0.983 (0.977,0.987)
	T3	3231	0.993 (0.990,0.996)	0.979 (0.974,0.984)
TG	T1	3103	0.997 (0.995,0.999)	0.983 (0.978,0.988)
	T2	3147	0.996 (0.994,0.998)	0.985 (0.982,0.990)
	T3	3256	0.992 (0.989,0.995)	0.977 (0.972,0.983)
		Cancer mortality		
Index	Tertile	Number of at risk	5-year survival (95%CI)	10-year survival (95%CI)
LDL-C	T1	3113	0.998 (0.996,0.999)	0.990 (0.987,0.994)
	T2	3152	0.997 (0.995,0.999)	0.987 (0.983,0.991)
	T3	3241	0.995 (0.993,0.998)	0.987 (0.983,0.991)
HDL-C	T1	3088	0.996 (0.993,0.998)	0.986 (0.981,0.990)
	T2	3147	0.998 (0.996,0.999)	0.991 (0.987,0.994)
	T3	3270	0.996 (0.994,0.998)	0.988 (0.984,0.992)
Non HDL-C	T1	3092	0.998 (0.997,1)	0.991 (0.987,0.994)
	T2	3181	0.997 (0.994,0.999)	0.988 (0.984,0.992)
	T3	3231	0.995 (0.993,0.997)	0.986 (0.982,0.990)
TG	T1	3103	0.998 (0.996,0.999)	0.992 (0.988,0.995)
	T2	3147	0.996 (0.994,0.998)	0.986 (0.982,0.991)
	T3	3256	0.996 (0.994,0.998)	0.987 (0.982,0.991)

(A) All-cause mortality.

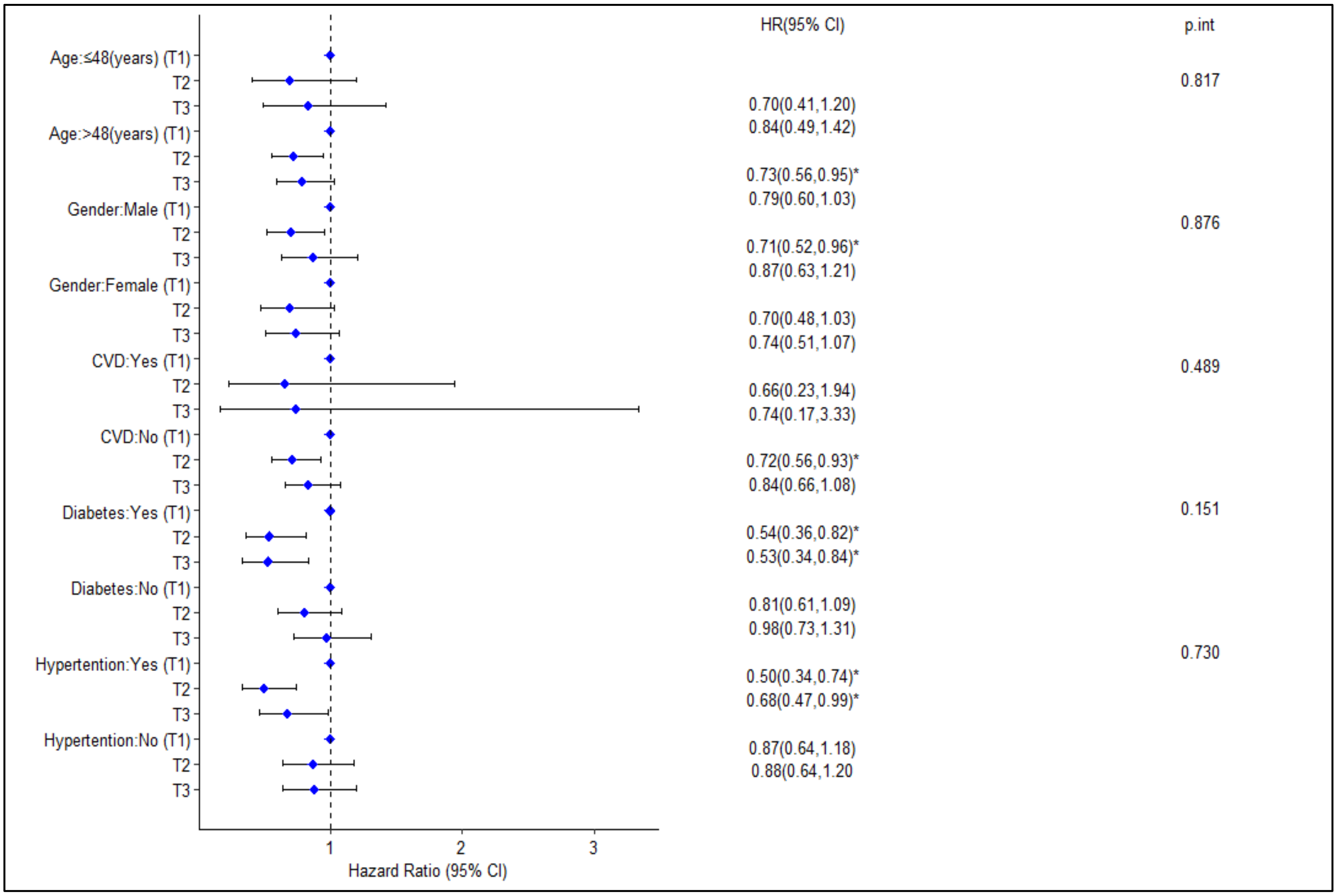

(B) CVD mortality

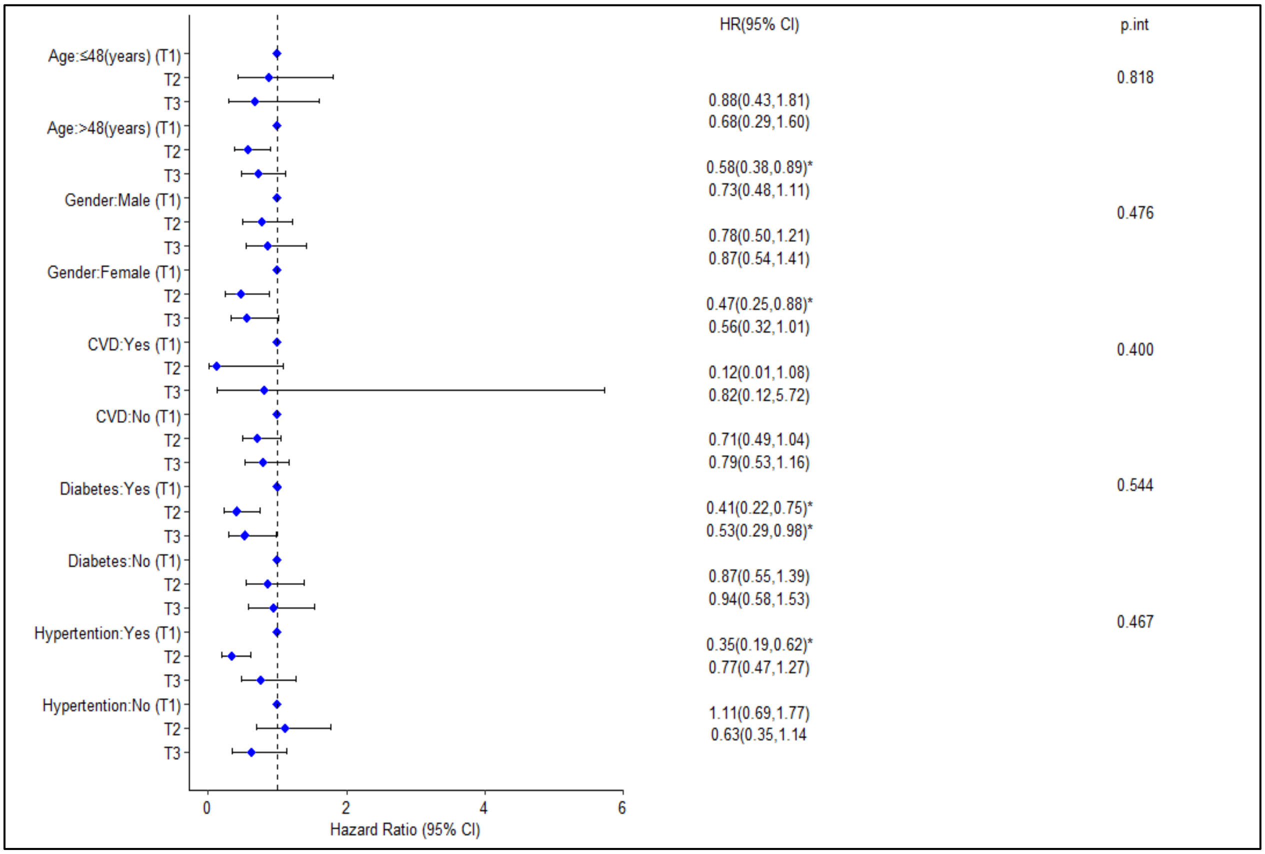

Figure S1. Subgroup analysis for the association of HDL-C level and all-cause (A) and CVD (B) mortality risk stratified by age, sex, Diabetes, Hypertension, and Dyslipidemia. Multivariable analyses were adjusted for age, sex, BMI, smoking status, diabetes, hypertension, dyslipidemia, CVD, job, marriage status, education level, and lipid-lowering drugs. p.int: P-value interaction

A) ROC curve of HDL-C to detect all-cause mortality in men

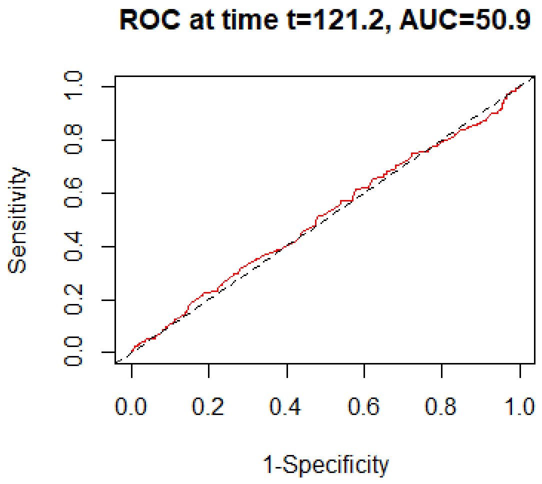

Sensitivity=51.43%   cut point=37.6

Specificity= 48.08%   Youden index=3.35

B) ROC curve of HDL-C to detect all-cause mortality in women

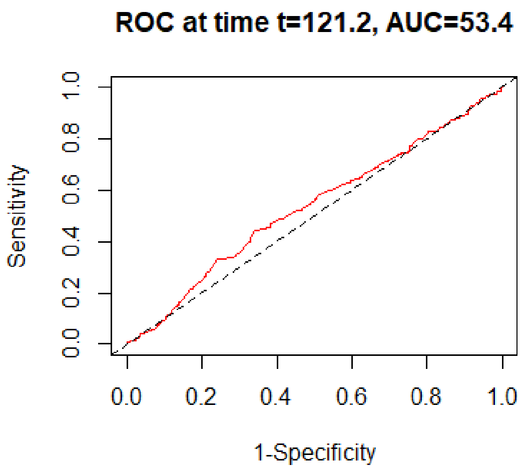

Sensitivity=44.12%   cut point=40

Specificity= 34.26%   Youden index=9.86

C) ROC curve of HDL-C to detect CVD mortality in men

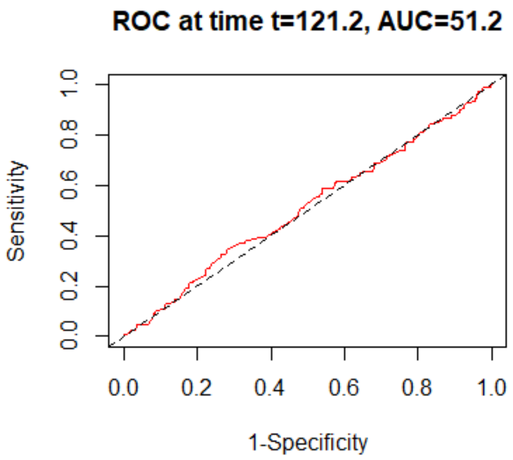

Sensitivity=37.35%   cut point=34.1

Specificity= 31.9%   Youden index=5.45

D) ROC curve of HDL-C to detect CVD mortality in women

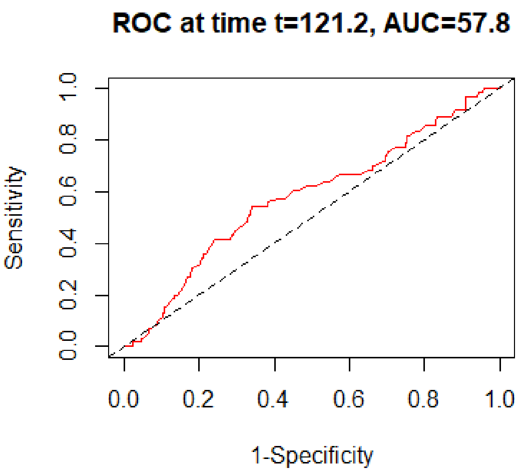

Sensitivity=54.24%   cut point=40

Specificity= 34.27%   Youden index=19.97

Figure S2. ROC curves of HDL-C adjusted based on Model3.
